# Comparing the DebugIT dashboards to national surveillance systems

**DOI:** 10.1186/1753-6561-5-S6-O1

**Published:** 2011-06-29

**Authors:** C Daniel, R Choquet, A Assele, F Enders, P Daumke, M-C Jaulent

**Affiliations:** 1 INSERM-AP-HP-Paris Descartes University, Paris, France; 2 UMRS 872 eq 20, INSERM, Paris, France; 3 Averbis, Freiburg, Germany

## Introduction / objectives

An important limitation of existing large-scale surveillance systems of infectious diseases is that they use mostly manual data collection processes and therefore usually deliver trends on an annual basis. The DebugIT project, funded by the 7^th^ EU Framework Programme, provides an access to heterogeneous clinical data sets of different European hospitals. We compared the DebugIT control capabilities to the process and results provided by the French surveillance system of infectious disease (Institut de Veille Sanitaire (InVS)) and the antimicrobial resistance surveillance study of the Paul-Ehrlich-Society (PEG).

## Methods

InVS currently controls every year multidrug resistant bacteria in 930 French healthcare facilities and Nosocomial Infection in 176 Intensive Care Units. PEG collects 240 isolates from each of 20-30 microbiology laboratories every three years. The DebugIT platform provides a scalable solution for executing real-time clinical queries over European data repositories about antibiotic resistance and antibiotic consumption.

## Results

Despite different methods for aggregating data and calculate incidence rates and antibiotic consumption (e.g. per 1,000 patient-days), the trends observed by national surveillance programs are similar to those reported retrospectively by the DebugIt platform. The detailed comparison is still ongoing.

## Conclusion

The use by European surveillance networks of platforms such as DebugIT platform is likely to enhance their ability for real-time identification of new trends in antibiotic resistance and/or antibiotic consumption. An interesting perspective is to connect DebugIT endpoints to general practitioner electronic medical records or private laboratory information systems in order to extend the surveillance to the community.

## Disclosure of interest

None declared.

